# The optimal window size for analysing longitudinal networks

**DOI:** 10.1038/s41598-017-13640-5

**Published:** 2017-10-17

**Authors:** Shahadat Uddin, Nazim Choudhury, Sardar M. Farhad, Md. Towfiqur Rahman

**Affiliations:** 1 Complex Systems Research Group, Faculty of Engineering & IT, The University of Sydney, Darlington, NSW 2008 Australia; 2 Department of Computer Science & Engineering, Bangladesh University of Engineering & Technology, Dhaka, 1200 Bangladesh

## Abstract

The time interval between two snapshots is referred to as the window size. A given longitudinal network can be analysed from various actor-level perspectives, such as exploring how actors change their degree centrality values or participation statistics over time. Determining the optimal window size for the analysis of a given longitudinal network from different actor-level perspectives is a well-researched network science problem. Many researchers have attempted to develop a solution to this problem by considering different approaches; however, to date, no comprehensive and well-acknowledged solution that can be applied to various longitudinal networks has been found. We propose a novel approach to this problem that involves determining the correct window size when a given longitudinal network is analysed from different actor-level perspectives. The approach is based on the concept of actor-level dynamicity, which captures variability in the structural behaviours of actors in a given longitudinal network. The approach is applied to four real-world, variable-sized longitudinal networks to determine their optimal window sizes. The optimal window length for each network, determined using the approach proposed in this paper, is further evaluated via time series and data mining methods to validate its optimality. Implications of this approach are discussed in this article.

## Introduction

Owing to the immense growth, proliferation, and availability of longitudinal network data and their inherently dynamic nature, it is imperative to develop a scalable and data-intrinsic architecture to analyse them effectively. An important aspect of this architecture involves the optimal/appropriate selection of sliding window sizes for temporally sampling longitudinal networks. A longitudinal network evolves over time among a set of actors. In recent years, the study of longitudinal networks has attracted considerable interest across a wide range of disciplines, including sociology^1,2^, communication and marketing science^3,4^, computer science^5^, scientometrics^6,7^, neuroscience^8^, cell biology^9^, epidemiology^10^ and ecology^11^, as researchers seek to determine the mechanism(s) underlying the processes of network formation, development and evolution over time. Researchers usually pay scrupulous attention to the design of their longitudinal studies and typically pay less attention to the temporal design of their studies. The latter refers to the timing and spacing of measurement collection^12^. One of the most important components of temporal design is the window size (i.e., the amount of time that elapses between occasions of consecutive measurements). All time-stamped network activities within each window are aggregated when conducting a longitudinal network data analysis. Window size selection is considered to be central to the design of any longitudinal research study; however, researchers often overlook this component.

The design of a longitudinal study involves the selection of a window size before or after data collection. In many cases, researchers must select the window size before conducting the underlying research. This practice, which is commonly followed in clinical cohort and psychological studies, involves frequently reporting of the window sizes chosen, but the reasons why a particular window size is chosen are not explained^13^. Conversely, the automated capturing of data from network sources allows researchers to choose different window sizes for research data analyses applied in their longitudinal studies^14^; for example, communication data from networked communities usually include timestamps. This attribute affords researchers the freedom to use different window sizes (e.g., a daily or weekly communication network) for their longitudinal studies based on the types of data involved. In studies that use communication data, researchers can also explore changes in results by considering different window sizes. However, to date, no research on longitudinal networks has adopted an approach to selecting a precise window size based on communication data. A poorly selected window size can cause researchers to draw inaccurate conclusions about the variables or hypotheses being studied. In a longitudinal study on smoking behaviour, for example, Collins and Graham^15^ found a positive correlation between smoking and peer smoking; however, the strength of the relationship was found to decrease as the window size increased.

When assigning a time span to the window of a longitudinal network, the underlying principle is that actors within the network must have sufficient time to initiate network processes, such as the formation and dissolution of ties. Actors in a longitudinal network usually exhibit different rates for different network activities (e.g., the formation of new ties or the dissolution of existing ties). For any given window with a specified time length, some actors exhibit higher levels of network activity than others; for example, an actor (Actor 1) in a longitudinal network might create only one new tie in a window, while another actor (Actor 2) in the same longitudinal network might forge five new ties in the same window. It may be the case that Actor 1 has exhibited a higher level of network activity at the very beginning of the next window, or Actor 2 has created all five new ties at the end of the present window. Conversely, Actor 1 might be very active in another window compared to Actor 2. A possible third scenario in relation to a given window size is that Actor 1 might reveal all its network activities (e.g., create 15 ties and delete 10 ties) within a longitudinal network in only one window while Actor 2 might engage in the same amount of network activities in five windows. This variability will significantly affect the involvement of actors in the evolution of the underlying longitudinal network. The size of a window can reduce the difference between network activities exhibited by the two actors. The aforementioned phenomenon (i.e., between Actors 1 and 2) is an example of possible scenarios applicable to any pair or group of network actors, and it is assumed that an appropriate window size should mitigate differences in network activities shown by a group of actors that exhibit a similar or different level of network activity during the evolution of an underlying longitudinal network. In consideration of temporal variations in actors’ network activities, Uddin, Khan, and Piraveenan^16^ found that actors in a longitudinal network exhibit two different types of dynamicities: positional dynamicity and participation dynamicity. The former captures variability in actors’ network positions over time, and its value is dependent on the social network analysis measure (e.g., in-degree centrality) used to quantify the network positions of actors. The latter represents the changing participation statistics of actors in a longitudinal network. In the present study, we hypothesise that the selected window size of a longitudinal network should ensure that the actors of that network exhibit a minimal level of variance in their positional dynamicity values. Simultaneously, the study shows that basic social network measures (e.g., centrality measures) can successfully delineate an actor’s positional dynamicity in longitudinal networks.

**Figure 1:**
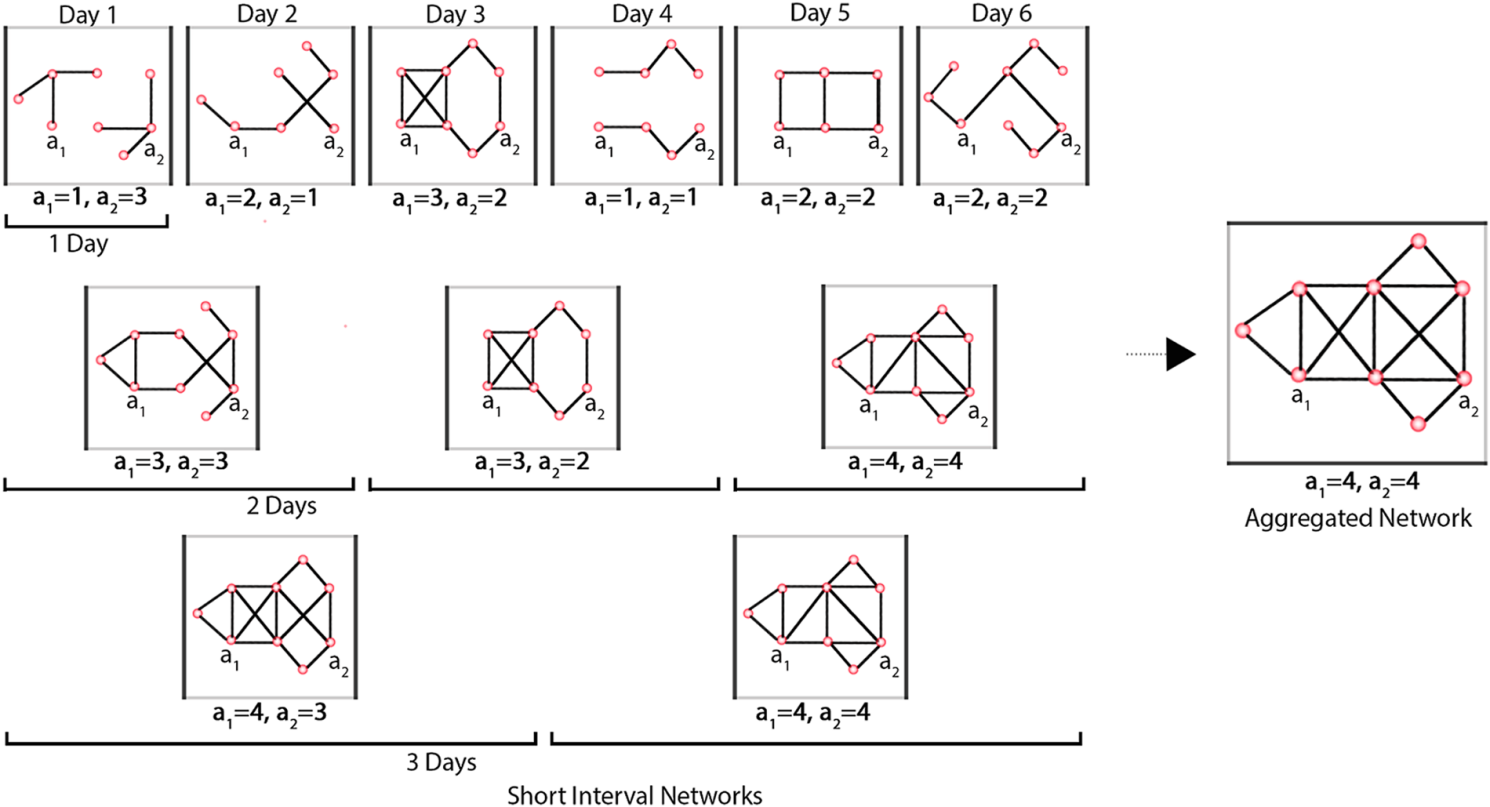
An abstract representation of a longitudinal network that evolved over six days. The visualisation demonstrates how a given longitudinal network can be represented as a collection of multiple short-interval, time-sliced networks constituting one aggregated network. The smaller network snapshots on the left side of the dotted arrow represent short-interval networks considering the temporal granularity of one, two and three days. The larger network on the right denotes their common aggregation. The actors have changing neighbourhoods depending on the sliding window size. Changes in the neighbourhoods of two actors (i.e., *a*_1_ and *a*_2_) in consideration of different window sizes (i.e., one, two and three days) are also shown. Numbers associated with two actors below each short-interval network denote neighbourhood degree values for the corresponding short-interval networks. Depending on the granularity of temporal slicing in defining short-interval networks, actors’ network centrality measures vary with respect to centrality measures observed in the corresponding aggregated network.

**Figure 2:**
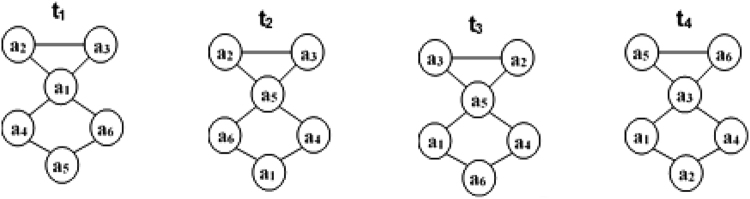
Snapshots of an abstract longitudinal network at four different time points: t_1_, t_2_, t_3_ and t_4_ (where t_4_ > t_3_ > t_2_ > t_1_). The network structures of these four snapshots are identical. For any network-level measure (e.g., density, centralisation and network diameter), the four snapshots give the same value. The variance of these values is then zero. However, actors in these four snapshots have different network positions (e.g., a_1_ and a_2_ are connected at t_1_ but not at t_2_). Any actor-level network measures (e.g., closeness centrality) can identify differences among these four snapshots. Hence, any approach that uses the variance of any network-level measure of different snapshots cannot suggest an appropriate window size for any longitudinal network with the same network-level structure for all snapshots.

The analysis of a given longitudinal network can produce different results for actor-level social network measures (e.g., network centrality) in consideration of different window sizes. Figure 1 shows this type of result. In this figure, two different actors (e.g., *a*_1_ and *a*_2_) have been found to have different degree values in network snapshots in consideration of different window sizes (i.e., one day to three days). Similar differences can be found when other social network measures (e.g., closeness centrality) are used to quantify actors’ network positions. An obvious question then arises – What is the optimal/appropriate window size for this longitudinal network? Few studies have attempted to address this question^17,18^. Related studies have considered the variance of network-level measures (e.g., density and centralisation) of different network snapshots but have not considered any actor-level measures as in the present study. Considering only network-level measures presents two problems. First, such studies cannot suggest an appropriate window size when only one network structure is involved but participating actors take different positions in different snapshots, as in such a case the variance of any network-level measure will be zero. Figure 2 shows an illustration of such a case. Second, such studies cannot always propose an appropriate window size for a given longitudinal network in consideration of any network-level measure^17^. The proposed approach employed in this study can overcome these two problems.

A given longitudinal network can also be analysed from different actor-level perspectives. For example, out-degree centrality values of actors in different windows can be used to explore how actors alter their network activities in a longitudinal network. In the same way, betweenness centrality values of actors can be used to explore how actors change their capacities to control flows of information between any pair of network actors over time in a longitudinal network. For this reason, different window sizes may be required when analysing a given longitudinal network from different actor-level perspectives.

This study outlines an empirical approach based on the concept of positional dynamicity in determining an appropriate window size when a longitudinal network is analysed from a specific actor-level perspective. The underlying aim of this approach is to determine a window size showing minimal variance among the network activities (captured by positional dynamicity) of all member actors in the evolution of an underlying longitudinal network. Minimal variance ensures that the suggested window size is neither too large for actors exhibiting high rates of network activity to exhibit a large volume of network activity nor too small for actors exhibiting slow rates of network activities to exhibit a minimal level of network activity. This approach is applied to four real-world longitudinal network datasets to determine their optimal window sizes from different actor-level perspectives. We then present evaluation methods based on time series analysis and unsupervised learning methods to validate the optimality of the identified sampling window size using the proposed approach. It is noteworthy that in longitudinal networks, the underlying time parameter is continuous where actor-oriented network activities unfolding in temporal steps are of arbitrarily varying lengths. In alignment with the existing literature^19^, this study also assumes that a network is observed at more than one discrete time point.

## Related work

The temporal sampling of a longitudinal network is often performed opportunistically^20^ depending on a wide range of factors. Timmons and Preacher^12^ identified some of these factors, including types of social networks, competing objectives, processes and measurements, planned time horizons of the respective study, the availability of funds, logistic and organisational constraints, the availability and expected behaviours of study participants and the desired accuracy and precision of study outcomes. Most theoretical and methodological approaches to defining optimal sliding windows of dynamic networks focus on the aggregation of links in time-window graphs^18^. This emphasis impacts the observation bias and the accuracy and significance of analyses, as dynamic network processes (e.g., the formation and dissolution of ties) can begin or end during inter-event periods^21^. Longitudinal networks are also analysed based on an assumption that more sampling generates better results^15,22^ or, in the case of randomised clinical trials, a sliding window size that maximises the efficiency of estimating treatment effects^23^. Frameworks of statistical analysis such as the *separable temporal exponential random graph model* ^24^ can also be used by relating the timing of network snapshots to the precision of parameter estimates.

The aforementioned approaches to determine an appropriate or optimal time window for analysing longitudinal networks suffer from their inherent limitations. For example, Timmons and Preacher^12^ found deteriorating outcomes from studies using more sampling windows and suggested that researchers consider the trade-off between precision and sampling time. On the other hand, statistical frameworks are parameter dependent and only work when applied to small networks with a few hundred actors. Recent studies focus on empirical analysis by comparing network statistics of temporal aggregations or graph metrics over time against threshold values in determining appropriate or meaningful sampling window sizes. For example, the Temporal Window in Network (TWIN) algorithm developed by Sulo, Berger-Wolf and Grossman^17^ analyses the compression ratio and variances in time series of graph metrics computed over a series of graph snapshots composed of temporal edges as a function of sampling window size. A window size is considered appropriate when the difference between variance and compression ratio for that window size is smaller or equal to a user-defined threshold. Soundarajan *et al*.^25^ defined another algorithm that identifies variable-length aggregation intervals by considering a ‘structurally mature graph’ that represents the stability of a network with respect to network statistics. A detailed study by Caceres and Berger-Wolf^20^ illustrates this windowing problem in reference to different formalisations and initial approaches to identifying the optimal resolution of edge aggregations, including their corresponding advantages and limitations.

## Positional Dynamicity

A longitudinal network includes different network snapshots observed at different points in time. These observed networks are called *short-interval networks* (SINs), and the accumulation of these SINs into a larger network is known as an aggregated network. In two consecutive SINs, the network involvement of an actor can be changed in two different ways. First, an actor can alter its neighbourhood connectivity within the two SINs and can thus change the network position captured by different network measures (e.g., degree, closeness and betweenness centrality). Second, an actor can change its presence; for example, an actor may be present in the *t* ^th^ SIN, leave the network at the *(t* + *1)* ^th^ interval and re-join the network at the *(t* + *2)* ^th^ interval. Thus, the dynamicity of an actor in a given longitudinal network has two components: (i) positional dynamicity (i.e., changes in actors’ network positions) and (ii) participation dynamicity (i.e., the statistics of an actor’s presence in different short-period networks).

According to social network analysis topology, a given longitudinal network must be analysed with respect to the temporal aggregation of links among its actors^26,27^. The quantification of different aspects of dynamicity as demonstrated by longitudinal networks is dependent on static and dynamic topologies of social network analysis^28^. Due to the temporal nature of SINs, dynamic topologies are exercised on temporal network snapshots, whereas the static topology is applied to an aggregated network. SINs can have different durations that principally depend on the underlying longitudinal network data. The accumulation of all SINs creates an aggregated network that forms a large cross-sectional network. Figure 1 also illustrates the different durations of short-interval networks and how they form an aggregated network. The rationale behind the concept of positional dynamicity and the reasoning for using this concept to identify optimal sampling window sizes are illustrated in Fig. 1. In this figure, network snapshots to the left of the dotted arrow represent the SINs of different durations (i.e., one, two and three days) and the larger network to the right denotes the aggregated cross-sectional networks. It is evident from the SINs of varying sizes that changes in network positions of actors are temporally dependent on the sampling resolution of SINs. Depending on the temporal granularity of SINs, an actor may either participate in network activities by forming links or may be concealed by its entire neighbourhood. Concurrently, the rate and volume of neighbourhood changes made by actors fluctuate in relation to the time and window size, which can be observed for the two actors (i.e., *a*_1_ and *a*_2_) shown in this figure. The concept of positional dynamicity was developed to quantify these temporal variations in consideration of dynamic and static social network topologies. Consequently, our objective is to define an optimal window length by considering the uniformity of positional dynamicities demonstrated by actors over time.

The positional dynamicity of a longitudinal network represents changes in the network positions of member actors across different SINs relative to their network positions in an aggregated network^16^. SINs may have different durations that mainly depend on the underlying longitudinal network data. An illustration of different durations of SINs and of how they form an aggregated network is also shown in Fig. 1. In assuming that a given longitudinal network is observed at *t*_1_, *t*_2_, *t*_3_ *… t*_*m*_ at different equal-time intervals (*t*_*m*_ > *t*_*m-1*_ > *t*_*m-2*_ > *…*. > *t*_2_ > *t*_1_ and t_m_ − t_m-1_ = t_m-1_ − t_m-2_ = …. = t_2_ − t_1_), where the aggregated network has *N* actors and where *m* SINs have *n*_1_, *n*_2_, *n*_3_ *… n*_*m*_ actors (where |n_1_Un_2_Un_3_U….Un_m-1_Un_m_| = *N*), Uddin *et al*.^16^ proposed the following equation for quantifying the positional dynamicity of a member actor of the given longitudinal network.

1 $$Po{D}_{i}=\frac{{\sum }_{t=1}^{m}\frac{|N{P}_{AN}^{i}-N{P}_{SIN(t)}^{i}|}{|N{P}_{AN}^{i}+N{P}_{SIN(t)}^{i}|}\times M(i,t)}{m}\times 100 \% \ldots \ldots \ldots \ldots \ldots \ldots $$

In Equation (1), *PoD*_*i*_ denotes the positional dynamicity demonstrated by the *i* ^*th*^ actor, $N{P}_{AN}^{i}$ denotes the network position measure (calculated by considering any actor-level social network measure, say closeness centrality) for the *i* ^*th*^ actor in the aggregated network, $N{P}_{SIN(t)}^{i}$ denotes the network position measure based on the same social network measure (i.e., closeness centrality) in the *t* ^*th*^ SIN for the *i* ^*th*^ actor, *M*(*i*, *t*) denotes the participation details of all actors in all SINs, and *m* denotes the number of SINs in the longitudinal network. A higher value of *PoD*_*i*_ for an actor indicates a higher level of variance in its network connectivity across different SINs of the underlying longitudinal network, and vice versa.

Subsequently, an equation for determining the positional dynamicity of a particular SIN was also proposed by these authors as follows:

2 $$Po{D}_{SIN(t)}=\frac{{\sum }_{i=1}^{N}\frac{|N{P}_{AN}^{i}-N{P}_{SIN(t)}^{i}|}{|N{P}_{AN}^{i}+N{P}_{SIN(t)}^{i}|}\times M(i,t)}{{n}_{t}}\times 100 \% \ldots \ldots \ldots \ldots \ldots \ldots $$

where *P*_*O*_ *D*_*SIN*(*t*)_ denotes the positional dynamicity of the *t* ^*th*^ short-interval network, *n*_*t*_ denotes the number of actors in the *t* ^*th*^ SIN, and *N* denotes the total number of actors in the aggregated network. A higher value for *P*_*O*_ *D*_*SIN*(*t*)_ indicates that actors presented in the *t* ^*th*^ SIN show higher differences between their network positions in that SIN compared with their network positions in the aggregated network.

In Equation (1), the value of $N{P}_{AN}^{i}$ for an actor is dependent on the underlying social network analysis (SNA) measure used to capture the network position of that actor. The value of $N{P}_{AN}^{i}$ can be different when the degree centrality measure is used to quantify the network positions of actors rather than the closeness centrality measure. Similarly, the value of $N{P}_{SIN(t)}^{i}$ is dependent on which SNA measure is used to quantify the network positions of actors. As both are used to measure *PoD*_*i*_ (i.e., on the right side of Equation (1)), the value of *PoD*_*i*_ is dependent on the SNA measure used to capture the network positions of actors. This value may be different when different SNA measures are used to capture the network positions of actors. The SNA measure developed for this study is a function of the degree, closeness and betweenness centrality of an actor in an individual SIN. This approach is used because these three centrality measures are the simplest and the most well defined and can successfully be used to quantify an actor’s connectivity, positioning, communication dynamics, influence and broadcasting capabilities, and importance in a network. Further, these measures are correlated. For example, an actor with high betweenness and low closeness centrality can monopolise links from a small number of actors to many others. Likewise, a high degree with low closeness centrality denotes an actor that is embedded in a cluster far from the rest of the network. The measure is defined as follows:

3 $${a}_{i}({g}_{t})={c}_{i}^{deg}({g}_{t})+{c}_{i}^{cls}({g}_{t})+{c}_{i}^{bet}({g}_{t}),\,i\in {v}_{t}\ldots \ldots \ldots \ldots \ldots $$

where *a*_*i*_(*gt*) denotes the SNA measure of actor *i* in a short-interval network *g* at time *t*. ${C}_{i}^{deg}({g}_{t})$ denotes the degree centrality, ${C}_{i}^{cls}$ denotes the closeness centrality, and ${C}_{i}^{bet}$ denotes the betweenness centrality of actor *i* in a short-interval network *g*. This measure ${a}_{i}({g}_{t})$ is used to quantify $N{P}_{AN}^{i}$ and $N{P}_{SIN(t)}^{i}$. Thus, the aggregation of degree, closeness and betweenness centrality measures of actors is used to quantify the positions of actors in SIN and aggregated cross-sectional networks.

## Proposed methodology for determining the window size

This section describes our approach to determining the window size of the underlying longitudinal network.

### Determining the window size

A two-step procedure was used to determine the window size of any given longitudinal network.

#### Step One

In Step One, we used Equation (1) to quantify actors’ positional dynamicity values for a given longitudinal dataset and we considered different lengths to define the SINs. The centrality measure defined in Equation (3) was used to calculate actors’ network positions for each SIN and in the aggregated network.

#### Step Two

For Step Two, we compared different sets of actors’ dynamicity values. In consideration of different lengths of SINs, we generated different sets of actors’ dynamicity values. A variance comparison approach was used to compare different sets of actors’ dynamicity values. For a group of numbers (e.g., a set of actors’ dynamicity values), the variance measured how far the numbers spread from their mean or average^29^. A higher level of variance among a set of numbers denoted a broad spread of numbers around the mean.

**Figure 3:**
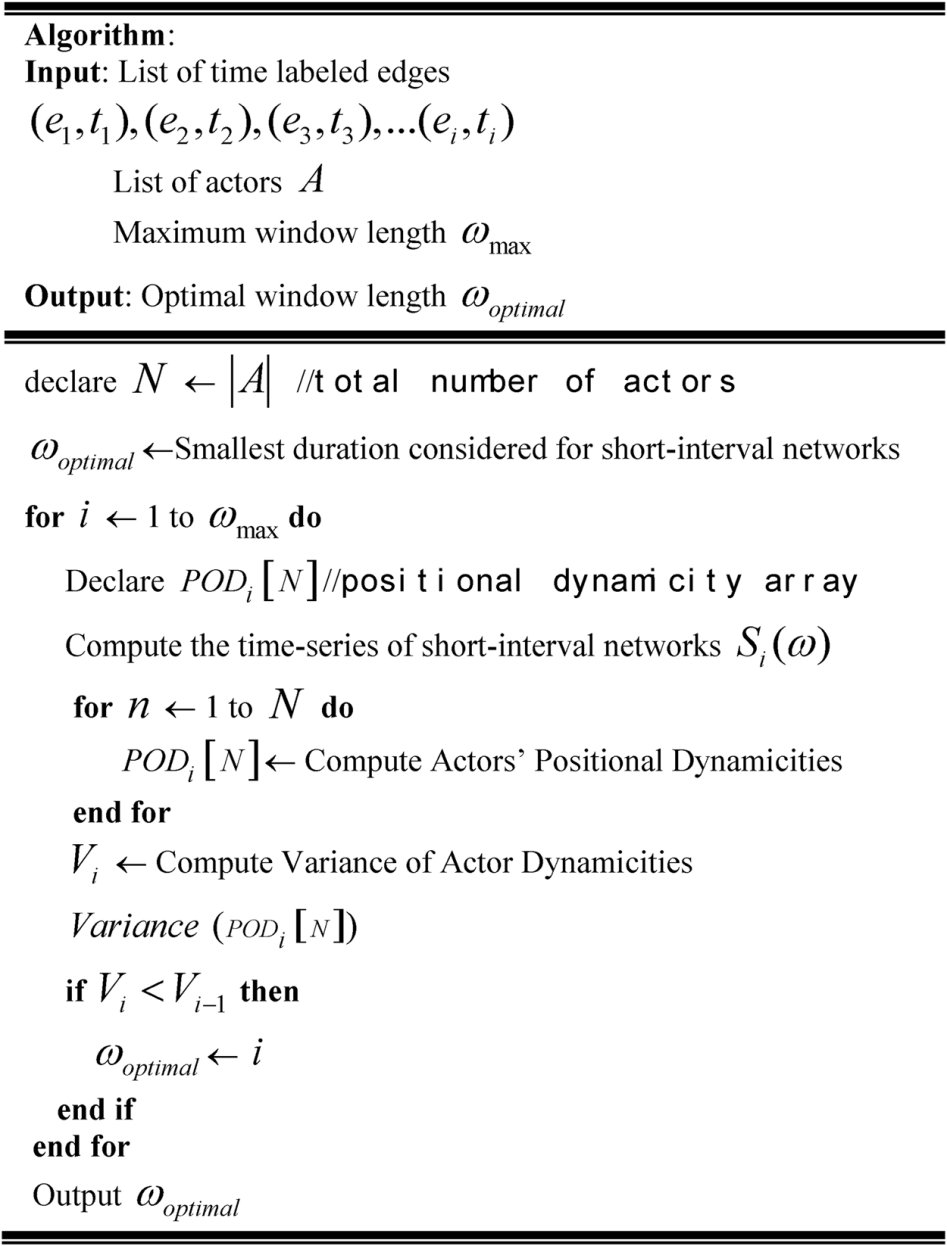
Algorithm for computing the optimal temporal sliding window size to analyse a given longitudinal network with time-labelled edges.

The corresponding length (used to define the SIN) was considered the right window size when it produced the lowest variance for the dynamicity values of all actors. Thus,

4 $${\rm{Window}}\,{\rm{size}}={\rm{S}};{\rm{for}}\,{\rm{which}}\,\text{Variance}\,({{\rm{AD}}}_{{\rm{i}}}^{{\rm{S}}})\,{\rm{is}}\,{\rm{the}}\,\text{minimum}\,{\rm{\ldots }}{\rm{\ldots }}{\rm{\ldots }}{\rm{\ldots }}{\rm{\ldots }}{\rm{\ldots }}$$

where $A{D}_{i}^{S}$ is the *Actor Dynamicity* value (i.e., positional dynamicity) of the *i* ^*th*^ actor (where *i* = 1, 2, …*n*) given that a length of *S* is considered in defining SINs and where there are *n* actors in the longitudinal network. The lowest variance for the dynamicity values denotes that actors exhibit the lowest degree of difference in positional dynamicity values over time in the underlying longitudinal network, which is the underlying principle of this research as discussed in the first section of this article. When actors’ positions in SINs and in the aggregated network are quantified by the degree centrality measure, for example, the lowest level of variance shows that actors exhibit a minimal level of difference among themselves in terms of variability in their degree centrality values over time in the underlying longitudinal network. Therefore, for a suggested window size, the lowest variance value confirms that (i) an active actor will not engage in a large number of network activities and (ii) an actor showing low rates of network activity will engage in a minimum volume of network activities. Figure 3 presents the algorithm that calculates the optimal window size for analysing longitudinal networks in consideration of the positional dynamicity of actors.

### Evaluation approach

This section describes evaluation approaches based on time series analyses and supervised learning methods used to validate the optimality of the identified window sizes for each dataset analysed in this study. Three different methods were used to validate the effectiveness of the proposed approach in identifying the optimal window length to sample a given longitudinal network: (i) an autoregressive integrated moving average (ARIMA) model, (ii) a time series anomaly detection method, and (iii) an unsupervised clustering method known as k-means clustering.

#### ARIMA Model

The dynamics of time-dependent complex social networks based on time series of network variables have attracted the attention of network science researchers. Time series analyses have been broadly adopted in network analysis methods (e.g., link predictions that model the underlying growth patterns of social networks). In a time series analysis, past observations of a time variable can be analysed to develop a model that predicts the future values of that variable. We use the ARIMA univariate time series method to model the temporal dynamicity of longitudinal networks. Under the ARIMA model, future values of a variable are determined from a linear combination of past values and errors. The model can be expressed as follows:

5 $$\begin{array}{ccc}{y}_{t} & = & {\theta }_{0}+{\phi }_{1}{y}_{t-1}+{\phi }_{2}{y}_{t-2}+\ldots \ldots +{\phi }_{p}{y}_{t-p}\\  &  & +{\varepsilon }_{t}-{\theta }_{1}{\varepsilon }_{t-1}-{\theta }_{2}{\varepsilon }_{t-2}-{\theta }_{q}{\varepsilon }_{t-q}\ldots \,\ldots \,\ldots \end{array}$$

where *y*_*t*_ = actual value; *ε*_*t*_ = random error at time *t*; and ${\phi }_{i}$ and ${\theta }_{j}$ are the coefficients, and *p* and *q* are the integers for the *autoregressive (AR)* and *moving average (MA)* polynomials, respectively. *ARIMA (p*, *d*, *q)* is an ARIMA model, where *p* is the number of autoregressive terms, *q* is the number of lagged forecast errors in the prediction equation and *d* is the number of non-seasonal differences required for stationarity.

Stationarity is important when developing a time series model, as the properties of a stationary time series do not depend on observation timestamps but only on white noise, which is Gaussian (random) in nature. Trends and seasonality affecting the value of time series at different times are two of the most important contributors to time series stationarity. Time series stationary can be achieved by differencing, which involves computing the differences between consecutive observations. This approach is used to stabilise the mean of a time series by eliminating trends and seasonality. Under the ARIMA model, parameter *d* represents the degree of differencing required to render a time series stationary. The higher the order becomes, the higher are the trends or seasonality present in the time series. Similarly, parameters *p* and *q* represent the lag order for both autoregressive and moving average processes, respectively, where these lag orders determine the degree of auto-correlation between time series values and associated error measures. For example, lag order *p* denotes that the time series value *v*_*t*_ at time *t* is correlated with value *v*_*t−p*_ at time (*t*−*p*). Interested readers are directed to the work by Hyndman^30^ for a detailed description of ARIMA. Considering this, *ARIMA*(0, 0, 0) is a white noise time series with no predictive pattern or trend components that requires no differencing and that shows zero correlation between time series values and associated error terms.

In considering temporal SINs in a longitudinal network as random networks, actor-level positional dynamicity represented by an individual SIN must be random without any correlation to the positional dynamicity generated by any previous SIN(s). Therefore, the time series of positional dynamicity demonstrated by the temporal sequence of SINs should be independent of any trend or seasonal component and should be random in nature. To this end, we determine a univariate time series with the help of Equation (2) that denotes the positional dynamicity of an individual SIN in a longitudinal network. Since different window lengths were considered in studying all network datasets of this study, we have different time series of SIN positional dynamicity. For these series, we attempt to determine the best-fit ARIMA model. As the underlying concept is that the dynamicity distribution across SINs of any length is free from trends and patterns (i.e., stationary series), the series that is most similar to *ARIMA*(0, 0, 0) is of the optimal SIN length.

#### Time Series Anomaly Detection

A time series is defined as a collection of observations of data items temporally collected through repeated measurements. A time series can be deconstructed into three components: (i) long-term variations or trends; (ii) systematic, seasonal or calendar-related movements; and (iii) out-of-control, irregular, and short-term fluctuations known as residuals. Seasonality generally involves regular, periodic, repetitive and predictable patterns, whereas the trend component is known as secular variations that denote long-term non-periodic variations. In time series anomaly detection, it is imperative to identify trend component(s) in time series that may introduce artificial anomalies into a time series^31^. In time series analyses, anomalies are denoted by point-in-time irregular data points. These anomalous data points can be global or local and positive or negative. Global anomalies extend above or below expected seasonality, and local anomalies appear within seasonal patterns and are always masked, rendering them difficult to detect. By contrast, positive anomalies represent point-in-time increases in observed values (e.g., number of tweets sent during a famous gaming tournament), while negative anomalies represent point-in-time decreases in observed values (e.g., number of service requests made to a server during a period of server malfunctioning). For this step, in addition to global and local anomalies within the time series of positional dynamicity values demonstrated by every SIN and computed from Equation (2), we identify positive and negative anomalies from cloud data using a novel anomaly detection technique^32^. It employs statistical learning approaches, time series decomposition and robust statistical metrics (e.g., median with Extreme Studentized Deviate (ESD)). This approach, known as the Seasonal Hybrid ESD, builds on the Generalised ESD test for detecting time series.

In using this approach, as was done when applying the ARIMA evaluation approach, we first calculated the positional dynamicity of SINs using Equation (2) and defined different time series in consideration of different window lengths. We then applied the Hybrid ESD to detect the percentage of anomalies in each time series and selected the time series with the fewest anomalies. The window lengths of SIN time series based on their positional dynamicity with minimum anomalies were used to find the optimal window length.

#### K-means Clustering

We applied an unsupervised learning approach to cluster actors based on actor-level dynamicity values for different window sizes computed using Equation (1). Using a popular unsupervised learning approach known as k-means clustering, we attempted to cluster actors into K groups based on their positional dynamicity values in consideration of the distribution of actor-level positional dynamicity as a Gaussian distribution. The k-means clustering algorithm uses initial estimates for randomly selected K centroids where each centroid defines one cluster and where each data point is assigned to its nearest centroid based on the distance function described below. The objective of k-means clustering is to minimise the intra-cluster variance known as the squared error function. The objective function is defined as -

6 $$J=\sum _{m=1}^{M}\sum _{n=1}^{N}\parallel {x}_{n}^{(m)}-{c}_{m}{\parallel }^{2}\ldots \ldots \ldots \ldots $$

where *M* denotes the number of clusters, *N* denotes the number of samples, *c* denotes the centroid for cluster *m*, and $\parallel {x}_{n}^{m}-{c}_{j}\parallel$ denotes the distance function. When the distribution of calculated actor dynamicity values is sparse (i.e., the variance is high or there are too many extreme values), and the range of actor dynamicity values is high, there will be more clusters than when the distribution exhibits low variance. Therefore, we first determine the optimal number of clusters through k-means clustering by considering the actor dynamicity values for different window lengths to identify the window size for which the distribution of actors’ positional dynamicity values has a minimal number of centroids or a lower number of clusters. Regarding the optimality of the number of clusters, the ultimate objective is to minimise the error measure, which is denoted by the total within-cluster sum of squares around cluster means as shown in Equation (6). We then attempted to find the total within-cluster variance or total within-cluster sum of squares (i.e., the square of the distance function in Equation (6)) of these clusters. The window size for which the total value of the within-cluster sum of squares or the value of the distance function in Equation (6) in k-means clustering over actor dynamicity values is the lowest is a potential candidate for the optimal window length.

According to the clustering approach, the identification of the optimal number of clusters is subjective and is dependent on the methods used to measure similarities among data points and parameters used for partitioning. Generally, clustering algorithms are designed for multivariate environments where datasets serve as a collection of features describing each data point. However, the popular heuristic k-means algorithm is unable to guarantee an optimal number of clusters for univariate data. As in this study we used one-dimensional data (i.e., actors’ positional dynamicity), we used the ‘*Ckmeans.1d.dp*’ algorithm developed by Wang and Song in^33^, which performs optimal one-dimensional k-means clustering via dynamic programming. Using this evaluation method, we first attempted to determine the optimal number of clusters in consideration of univariate actors’ positional dynamicity distributions using different window sizes. Second, for each window size, we calculated the within-cluster sum of squared distances. For each network dataset, the window size with the lowest values for both quantities (i.e., optimal number of clusters and within-cluster sum of squared distances) was determined to be the optimal window size.

## Longitudinal network datasets

As the proliferation of dynamic network data occurs across a myriad of real-world domains, the network science community is observing an abundance of longitudinal network datasets. For data collection purposes, we used ‘Network Repository’, the oldest and largest interactive repository of network datasets^34^. Under the dynamic dataset category, there are 22 network datasets, and from these, we extracted four datasets. The first data set includes network data drawn from a Facebook-like social network (UCI Network) originated from an online community of students at the University of California, Irvine (nodes represented students within the community, and edges represented messages sent between the students). The second undirected network dataset includes human contact data for 100 students of the Massachusetts Institute of Technology collected as part of the Reality Mining experiment performed in 2004 and as part of the Reality Commons project (MIT network). The third dataset includes internal email communications (Email network) among employees of a mid-sized manufacturing company (nodes represented employees, and edges represented individual emails sent between two employees). The final dataset includes a real Facebook friendship network (Facebook network), for which Facebook users were actors and friendship relations among users were links. We called these four longitudinal networks ‘UCI network’, ‘MIT network’, ‘Email network’ and ‘Facebook network’ for the sake of brevity.

Each longitudinal network dataset was split into smaller temporal graphs that considered different time slices to generate SINs for consideration. As discussed in the first section, we assume in this study that a given longitudinal network consists of smaller SINs that unfold at discrete time points and that all links in our four network datasets are date labelled. We thus consider nine different temporal granularities as our time unit. These include seven units for one-seven days and two units for 15- and 30-day intervals. For the sake of our analysis, short-interval networks of one day are denoted daily SINs, those of seven days are denoted weekly SINs, those of 15 days are denoted fortnightly SINs and those of 30 days are denoted monthly SINs. Table 1 shows the basic statistical features of the four datasets, including durations in dates and the number of temporal graphs or SINs under different time slices that split the time-stamped data such that each SIN includes links within a particular slice. The bottom of the table details the total number smaller sub-networks or splits comprising the total duration of the datasets in relation to a smaller duration of days. For example, the Email network had a total network duration of 272 days, of which 238 days had at least one edge defined for a day that was not a self-loop (i.e., when the source and target node of a link are the same).

**Table 1:** Basic statistics for the four longitudinal network datasets used in this study.

**Item**	**UCI Network**									**MIT Network**								
Nodes	1899									96								
Edges	59,834									1,086,403								
Date	*Start Date*				*End Date*					*Start Date*				*End Date*				
	16/04/2004				26/10/2004					05/05/2005				14/09/2004				
Window Size (days)	1	2	3	4	5	6	7	15	30	1	2	3	4	5	6	7	15	30
No. of SINs	193	97	65	49	39	33	28	14	8	234	117	78	59	47	39	34	16	8
	**Email Network**									**Facebook Network**								
Nodes	167									13,895								
Edges	82,875									206,668								
Date	*Start Date*				*End Date*					*Start Date*				*End Date*				
	02/01/2010				30/09/2010					14/10/2004				30/06/2007				
Window Size (days)	1	2	3	4	5	6	7	15	30	1	2	3	4	5	6	7	15	30
No. of SINs	238	133	91	68	55	46	39	19	10	901	474	322	243	197	164	141	49	25

The number of short-interval networks (SINs) denotes the total number of short-interval networks generated by dividing the whole dataset into smaller temporal networks using different window sizes or durations.

## Results derived from the sampled datasets

In this section, we present the results of our experiment on four longitudinal network datasets.

### Optimal Window Size

We first perform a variance analysis of actors’ positional dynamicity values based on nine window sizes for four datasets. In Table 2, we present the results of our variance analysis. In this table, we show that for the Email and MIT networks, the optimal window size is 30 days. The second best window size for these two longitudinal networks is 15 days. Therefore, we conclude that monthly or fortnightly short-interval networks suffice when analysing these dynamic networks. By contrast, for both the Facebook and UCI networks, the proposed approach identifies a duration of one day as the optimal sliding window size for generating network snapshots. The second best optimal window size for the Facebook and UCI networks is two days, as identified by our variance analysis of actors’ positional dynamicity values.

**Table 2:** Variances of positional dynamicity values for all actors based on nine different window sizes.

Dataset	Window Size (days)								
	1	2	3	4	5	6	7	15	30
UCI Network	0.0020	*0.0031*	0.0035	0.0037	0.0041	0.0041	0.0044	0.0045	0.0041
MIT Network	0.0102	0.0115	0.0118	0.0116	0.0114	0.0110	0.0105	*0.0097*	0.0079
Email Network	0.0187	0.0186	0.0228	0.0210	0.0194	0.0178	0.0166	*0.0121*	0.0092
Facebook Network	0.0001	*0.0004*	0.0007	0.0010	0.0014	0.0019	0.0023	0.0054	0.0097

The cell with underlined texts denotes the lowest variance (i.e., corresponding window size is the optimal one) and the cell with italic texts denotes the second lowest variance for each longitudinal network examined in this study.

### Optimal Window Size Validation

**Figure 4:**
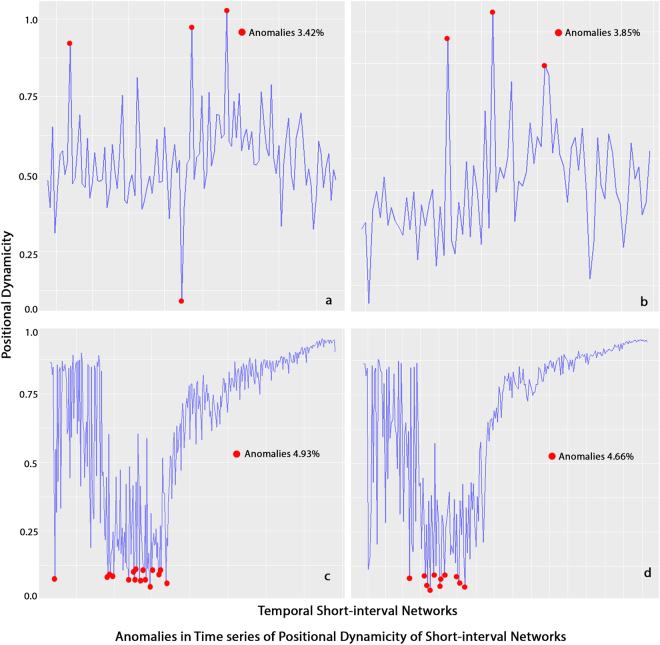
Visual representations of percentages of anomalies present in time series of positional dynamicity values for every short-interval network (SIN). Time series were built for all SINs in the MIT (top) and Facebook networks (bottom) based on window sizes of two (*a* and *c*) and three days (*b* and d) as sliding window sizes.

For this step, we validate the identified optimal window sizes of the different longitudinal network datasets. This step is performed by determining the best fit ARIMA model and the percentage of anomalies in time series of positional dynamicity values of SINs, and by identifying minimum total within-cluster variance levels (i.e., sum of squared errors) among the optimal number of clusters via k-means clustering in consideration of the positional dynamicity of actors in SINs. Table 3 shows these validation results. In this validation phase, we used two R packages: ‘forecast’^35^ for ARIMA validation and ‘AnomalyDetection’^31^ for time series anomaly detection. The latter is capable of detecting percentages of anomalies present in univariate time series, including the directionality (i.e., positive, negative or both) of anomalies. This package in using the Seasonal Hybrid Extreme Studentised Deviate (S-H-ESD) method can detect the maximum number of anomalies present as a percentage of data. This percentage value was set to five. We thus considered time series with at most five percent anomalies. In Fig. 4, we provide visual representations of the percentage of anomalies present in time series of positional dynamicity values as demonstrated by SINs of the MIT (top row) and Facebook networks (bottom row) using the window sizes for two days (Fig. 4(a,c)) and three days (Fig. 4(b,d)) as sliding window sizes. For k-means clustering, we used the associated R package^33^.

**Table 3:** Results justifying the optimal window size (in days) of nine window sizes as determined by the proposed approach applied to four longitudinal networks.

UCI Network									
Validation tests	Window Size (days)								
	1	2	3	4	5	6	7	15	30
1. Best Fit ARIMA	(0,1,1)	(0,1,1)	(0,1,1)	(0,1,0)	(0,1,0)	(0,1,0)	(0,1,0)	(0,1,0)	(0,1,0)
2. Time Series Anomaly (%)	0	0	0	0	0	0	0	3.15	3.57
3. k-means clustering									
*3.1 Optimal number of clusters*	8	8	8	8	9	8	7	8	8
*3.2 Minimum Total Within-cluster Variance*	0.084	0.127	0.155	0.151	0.121	0.163	0.226	0.170	0.150
MIT Network									
1. Best Fit ARIMA	(1,0,0)	(2,0,2)	(0,1,1)	(0,1,1)	(0,1,1)	(0,1,0)	(0,1,0)	(0,1,0)	(0,1,0)
2. Time Series Anomaly (%)	0.85	0.85	1.28	0	0	0	0	0	0
3. k-means clustering									
*3.1 Optimal number of clusters*	1	1	1	2	1	2	2	2	2
*3.2 Minimum Total Within-cluster Variance*	0.969	1.092	1.119	0.284	1.082	0.256	0.255	0.252	0.225
Email Network									
1. Best Fit ARIMA	(1,0,0)	(1,1,1)	(2,1,3)	(1,1,1)	(0,1,1)	(0,1,1)	(0,1,1)	(0,1,0)	(0,1,0)
2. Time Series Anomaly (%)	4.62	4.51	4.40	4.41	3.64	4.35	2.56	0	0
3. k-means clustering									
*3.1 Optimal number of clusters*	6	4	4	4	3	3	3	3	3
*3.2 Minimum Total Within-cluster Variance*	0.059	0.196	0.236	0.222	0.400	0.357	0.344	0.260	0.214
Facebook Network									
1. Best Fit ARIMA	(3,1,3)	(0,1,1)	(4,1,4)	(2,1,2)	(0,1,1)	(2,1,2)	(4,1,0)	(0,1,0)	(0,1,0)
2. Time Series Anomaly (%)	0	0.87	0.85	0.56	0.68	0.83	0.96	0.96	4.00
3. k-means clustering									
*3.1 Optimal number of clusters*	8	8	9	9	9	9	9	8	9
*3.2 Minimum Total Within-cluster Variance*	0.026	0.081	0.115	0.172	0.232	0.301	0.363	1.032	1.442

The evaluations involved three types of validation tests: the best-fit ARIMA model, the percentage of time series anomalies in time series of the positional dynamicity of SINs of nine different lengths and the total within-cluster variance or total within-cluster sum of squares of the optimal number of clusters determined from k-means clustering on a distribution of actor positional dynamicity values.

**Figure 5:**
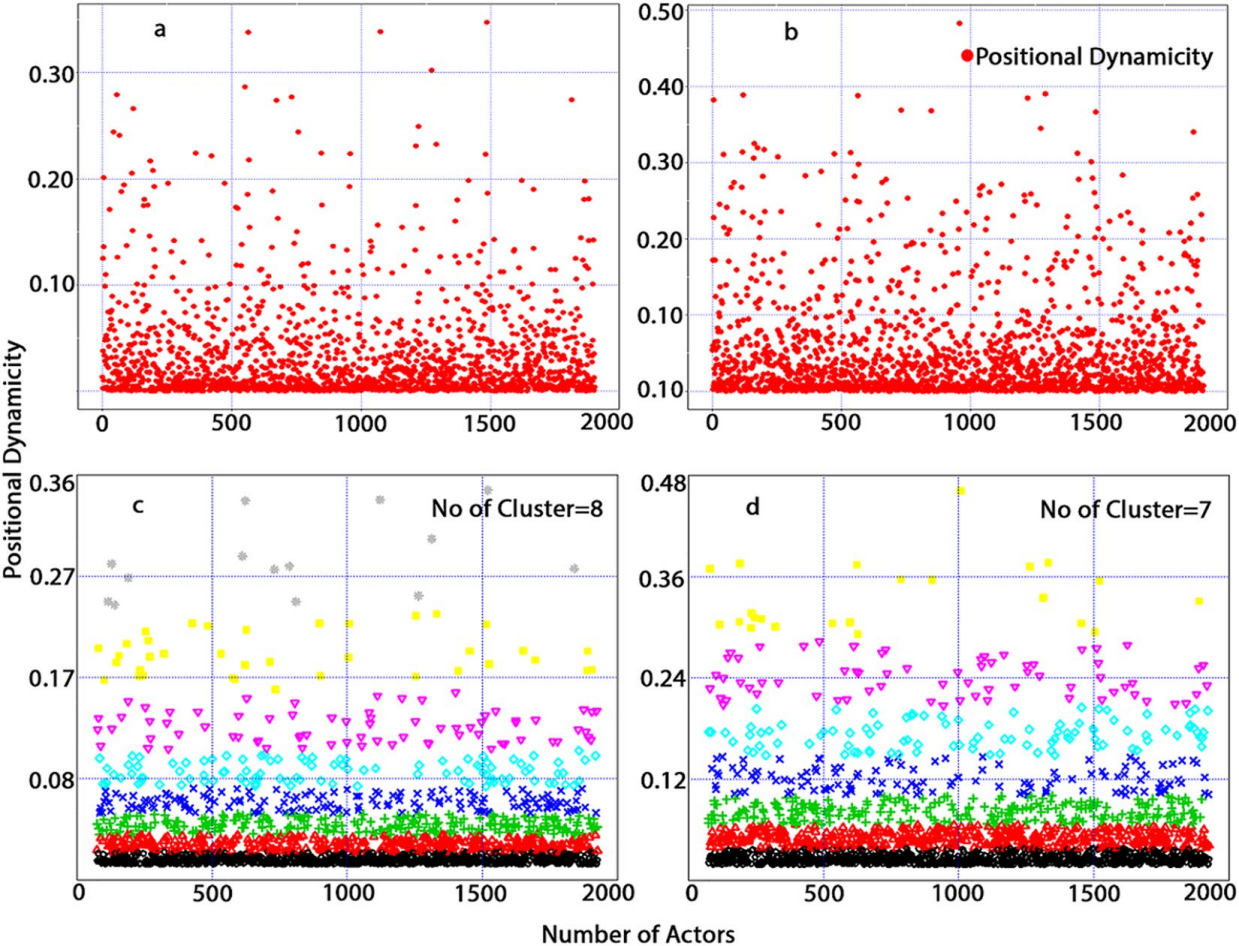
Distribution of actors’ positional dynamicity values and corresponding clusters in the univariate k-means clustering of the UCI network based on window sizes of one day (*a* and *c*) and seven days (*b* and *d*). In the top row, two graphs present the distribution spread with the number of actors along the x-axis and corresponding positional dynamicity values along the y-axis. Along the bottom row, corresponding clustering information is provided based on k-means clustering, where for a one-day window size, the number of clusters is eight, while for a window size of seven days, the number of clusters is seven.

From Table 3, it is apparent that the optimal window size of one day for the UCI network, as determined by the approach proposed in this study and presented in Table 2, passes the validation test based on time series anomaly detection. With respect to the optimal number of clusters of k-means clustering over the positional dynamicity values of actors, a one-day window size produced the second best result (i.e., eight) relative to the minimum number of clusters (i.e., seven) found when considering a window size of seven days; however, considering the minimum within-cluster variance, the identified optimal window size outperformed other window sizes in the UCI network. In Fig. 5, we provide a visual representation of the spread of positional dynamicity (i.e., top row of the figure) demonstrated by actors in the UCI network when SIN lengths of one (i.e., left side of the figure) and seven days were considered (i.e., right side of the figure) to better understand the above finding (i.e., number of clusters vs. total within-cluster variance). The bottom two figures show clustering information generated from the Ckmeans.1d.dp k-means algorithm for univariate data where the number of clusters for a seven-day window length is seven, whereas the number of clusters for the optimal window length (i.e., one day) is eight. While the total number of clusters is not the lowest for the optimal window size, it maintains a trade-off by increasing the number of clusters without forfeiting the error rate. Thus, we observe lower within-cluster variance levels from the resulting clustering data of actors’ positional dynamicity. With respect to the ARIMA validation, the optimal window size generated the second best score.

For the MIT network, we found 30 days to be the optimal window size followed by fifteen days. A window size of one day was found to be the third best and a close contender, as is shown in Table 2. With respect to the best-fit ARIMA model, the top two optimal window sizes fit well, but we found trends (i.e., *d* = *1* in the ARIMA model) in time series of positional dynamicity values of SINs considering fortnightly and monthly SINs. Conversely, for daily SINs, we found no trends. Regarding the percentage of anomalies present in the same time series of positional dynamicity values for SINs, the optimal window size passed the validation test. Finally, for the validation test based on k-means clustering, we observed behaviours similar to those observed from the UCI network. Despite not being the lowest with respect to the number of clusters using k-means, the total within-cluster error rate (i.e., 0.225) is the lowest of the window sizes.

For the Email network, while the ARIMA test results show no correlations between time series values with respect to autoregressive and moving average lag orders, the model shows trends in time series positional dynamicity as demonstrated by SINs from an optimal window length of 30 days. Notwithstanding, for the Email network, the optimal window length passes time series anomalies. According to the clustering test results, for this dataset, we find that window size of one day presents the lowest level of total within-cluster variance but the highest number of clusters. Considering the trade-offs noted above, we find that the optimal window length presents the lowest total within-cluster variance of the window sizes and the lowest optimal number of clusters in k-means (i.e., three for the Email network).

For the Facebook network dataset, the optimal window length of one day was unsuccessful in qualifying for the best fit ARIMA model, as it showed higher values for autoregressive, differencing and moving average polynomials relative to the other window sizes. However, for this network, the optimal window length successfully outperformed the other window sizes in consideration of the rest of the validation tests despite demonstrating higher order parameter values for the ARIMA model.

This study suggests to consider a duration, which shows lowest variance in actors’ dynamicity values, as the optimal window size if it passes majority of the validation tests considered in this study; otherwise, consider the next best possible candidate (showing the second lowest variance) for the optimal window size and conduct the same validation tests. Overall, considering the trade-off between the optimal number of clusters and the minimum total within-cluster variance, both UCI and Facebook datasets passed two validation tests (out of three) for the window sizes suggested by the proposed approach of this study. MIT and Email datasets passed all three validation tests for the window sizes suggested by the proposed approach with the similar trade-off consideration.

### Comparison

A few ways of determining the optimal window size for the analysis of a given longitudinal network have been presented in the literature. Among them, commonly used approaches include the TWIN^17^, GraphScope^36^ and Fourier Transform Analysis^37^ methods. The first approach has been found to outperform the other two^17^. For this reason, we compare the proposed approach with the TWIN. For SINs of a longitudinal network, the TWIN calculates the network-level statistics (e.g., network density, centralisation and network diameter) of SINs and their variance. It then computes the compression ratio of the string representation of network-level statistics under consideration. The window size for which the difference between the variance and compression ratio is minimal (or less than a user-defined ‘goodness measure’) is the appropriate window size for analysing the underlying longitudinal network.

**Figure 6:**
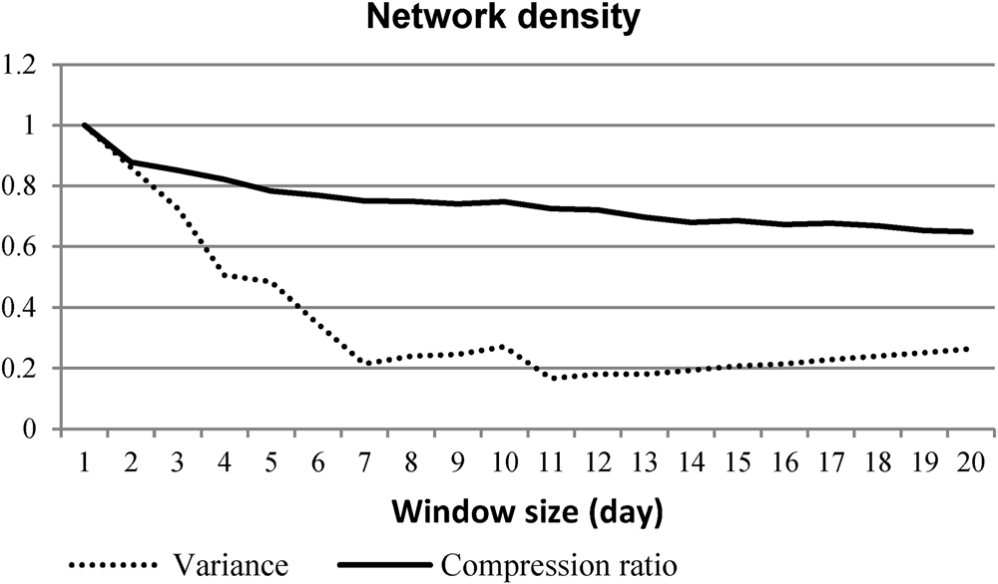
The compression ratio and variance of network density for different window sizes for the Facebook network. A window size of one day generates the lowest difference between them. Hence, for this network, the optimal window size is one day for the network-level metric of density. The proposed approach of this study also suggests an optimal window size of one day for this network.

With respect to network-level measures of density (the proportion of the number of edges present in a network compared to the potential number of all edges among network actors) and the clustering coefficient (the number of triangles over the potential number of triangles in a network), the TWIN and the approach proposed in this study propose the same window size (one day) for analysing the Facebook network. Similarly, for the ‘number of connected components’ (a set of nodes that are mutually reachable by paths in the network) metric the proposed approach suggests the same window size (one day) as the TWIN for the UCI network. For both the MIT and Email networks, the proposed approach suggests the same window size as the TWIN for a user-defined ‘goodness measure’ of 0.3 but only for network-level statistics of the ‘size of the largest clique’. In a network, a clique is a group of nodes that can directly reach one another. Figure 6 presents an example based on the network density measure, wherein the TWIN proposes an optimal window size of one day for the Facebook network. The proposed approach of this study also suggests a window size of one for the same network.

Although for each network-level measure, the proposed approach does not suggest the same window size as the TWIN, the proposed approach can overcome two of the main limitations of the TWIN (as outlined in the first section). First, it can propose an optimal window size for all SINs with the same network-level structure but with different actor network positions (as is shown in Fig. 2). In such cases, the TWIN is not applicable as the variance will be zero. Second, the proposed approach can suggest an optimal window size for any longitudinal network, whereas for a given longitudinal network the TWIN cannot make such suggestions considering any network-level measure used to quantify SINs^17^.

## Discussion and Conclusion

For dynamic or longitudinal networks, it is necessary to identify correct, appropriate or optimal levels of aggregation granularity to bin any stream of timestamped links to discern meaningful data and to understand the dynamics of such networks. As identified by Fish and Caceres^38^, researchers have defined this problem differently (e.g., change point detection, time scale detection, oversampling correction, temporal resolution inference, aggregation granularity detection or windowing selection). This identification of correct window lengths strongly impacts structural analyses, the efficacy of network mining and dynamics demonstrated by networks^39–41^. Using temporal granularity levels that are too coarse or fine can conceal or fail to unravel critical data on network dynamics and can limit understanding of the structure of underlying interactions. Furthermore, appropriate temporal binning decisions made in dynamic networks enable one to distinguish among noisy, local and critical temporal orderings.

In the literature, there is a lack of standardisation in regards to the selection of optimal window sizes for analysing longitudinal networks, and often, this task is left to the arbitrary choices of scholars depending on the experimental contexts or requirements of a corresponding study. In some cases, selection is also determined based on data collection processes, which is impractical. Researchers have also attempted to exploit network-level structural properties across temporal network snapshots to identify the appropriate window length, as discussed in the ‘related work’ section. We also describe deficiencies of such measures in the ‘comparison’ section of this paper. We thus propose an approach that can be used to determine appropriate window sizes for the analysis of any longitudinal network in relation to different actor-level perspectives. The approach is based on the concept of actor-level dynamicity, which quantifies changes in actors’ network involvement levels (in terms of network positions) during the evolution of an underlying longitudinal network. In detecting optimal window lengths, we first defined a positional dynamicity measure that quantifies changes associated with an actor’s structural positions in networks over time. We used a combination of three well-defined centrality measures to measure the positional dynamicity values of actors. These centrality measures have long been exploited in network science not only to quantify actors’ network activities but also to define actor prominence, communicability and reachability. We also used four real-life longitudinal network datasets, where each network is split into different SINs based on window lengths of one to seven days, 15 days (fortnight) and 30 days (monthly). To determine optimal window lengths from these nine sampling resolutions, we compared the variances of nine sets of actor dynamicity values. The window length with the lowest levels of variance in actor dynamicity distributions was found to be the appropriate sampling window for analysing longitudinal networks because the minimum variance ensures that the suggested window size is neither too large for actors with high levels of network activity to exhibit large levels of network activity nor too small for actors exhibiting low levels of network activity to exhibit a minimal level of network activity. We also evaluated the optimal window sizes identified through validation tests involving time series analysis and unsupervised learning methods, which justify the optimal window length derived from the proposed approach. For the MIT network, the optimal window length successfully passing the validation tests was identified as 30 days (i.e., monthly). For the Email network, we found a similar optimal window size of 30 days from the validation tests. For UCI and Facebook networks, the daily window size was found to be optimal and was successfully approved through the validation steps.

The method proposed in this manuscript can also be applied to unequal spacing in time intervals. In such cases, an underlying longitudinal network must be divided into several shorter longitudinal networks. Each of these short networks can then be considered a separate longitudinal network. The approach proposed in this manuscript can be applied to each of these short networks to find corresponding best window sizes. For example, a longitudinal network of 52 weeks can be considered two shorter longitudinal networks: one from 1 to 26 weeks and another from 27 to 52 weeks. After the approach proposed in this study is applied, the best window size for the first shorter longitudinal network can be determined as two days, while that for the second can be determined as five days.

This article quantified actors’ positions in SINs by combining three centrality measures. Thus, the suggested window size (based on the positional dynamicity) has a single value for different SIN lengths. When more measures are considered in quantifying actors’ positions in SINs (e.g., considering three centrality measures separately), suggested window size values vary because actors in a longitudinal network can present different positional dynamicity values for different social network analysis measures. Some actors may exhibit higher levels of dynamicity in one network measure but lower levels of dynamicity for another network measure. Thus, the suggested window size can be different for the same longitudinal network when different network measures are used to calculate the actors’ network positions. An obvious question then arises: when should one consider a particular window size from a number of suggested values in analysing a given longitudinal network? The context of any underlying longitudinal network can be considered when determining a window size from a list of alternatives. For example, noting that degree centrality represents the activity and popularity of an actor in a network^42^, the window size suggested when degree centrality has been used to quantify actors’ network positions in SINs can be considered in the analysis of political longitudinal networks, as levels of activity and the acceptability of member actors are very important in this type of network^43^. In other contexts, where several centrality measures play an important role in actors’ structural attributes (e.g., an organisational communication network), centrality measures may be combined to quantify actors’ structural positions.

In applying the proposed approach to determine appropriate window sizes, we used electronically collected longitudinal network datasets (i.e., a social media dataset and email communication dataset). These datasets were collected at different points in time so that comparisons could be drawn among different actor-level dynamicity values derived from different SIN lengths. In a longitudinal analysis, this approach can easily be used to determine the appropriate window size of social media and electronically collected communication data. In relation to the data collection design of any future survey-based longitudinal study, the proposed approach for determining the window size cannot be replicated directly; however, the proposed approach can be used to determine window sizes in such studies. Under this approach, all previously conducted survey-based longitudinal study data for any given research context should be collected first. The research approach proposed in this study could then be applied to such datasets to approximate the optimal window size of any future survey-based longitudinal study within a similar research context.

This article addresses a classic network science problem: how to determine the right window size for a longitudinal network analysis. Our approach differs from those applied in other studies in regards to its simplicity and applicability; it does not need to maintain ground truth (i.e., more sampling is better) and is not dependent on parametric distributions. Its simplicity lies in its introduction of simple centrality measures that are computationally efficient. By contrast, matrix and tensor-based methods cannot be applied to real-time large networks owing to their computational complexity and time requirements^44^. Furthermore, unlike other methods described in the ‘related work’ section, the proposed method uses metrics related to the network structural evolution of actors (the central constituents of a network) rather than network or graph metrics. This study can be extended further by considering other actor-level centrality measures (i.e., k-coreness) in defining actor positional dynamicity and temporal granularity (i.e., hours, minutes, fortnight, and year) to define the time series of network snapshots.
